# Dermomina^®^ clay achieves high closure rates in non-infected diabetic foot ulcers

**DOI:** 10.3389/fendo.2026.1742928

**Published:** 2026-02-11

**Authors:** Angiolina Camilo Reynoso, Angel Diaz Beltre, Albert Figueras

**Affiliations:** 1 Clinical Research Department, Instituto Nacional de Investigación de Enfermedades Infecto-Contagiosas (INIEICONT)/MESCYT, Santo Domingo, Dominican Republic; 2 Faculty of Health Sciences, Universidad Autónoma de Santo Domingo, Santo Domingo, Dominican Republic; 3 Independent Consultant on Medicines Use and Safety, Barcelona, Spain

**Keywords:** cost-effective healthcare, clay therapy, diabetic foot, skin ulcer treatment, wound healing

## Abstract

**Introduction:**

Diabetic foot ulcers (DFUs) remain a major global health burden, frequently leading to lower limb amputations, reduced quality of life, and premature mortality. Despite advances in multidisciplinary care, healing outcomes remain suboptimal, particularly in infected ulcers and in low resource settings. There is a need to explore accessible and cost effective adjunctive therapies that may support wound healing and improve outcomes.

**Methods:**

A pre experimental observational study was conducted at two wound care units in the Dominican Republic between March 2022 and October 2024. Dermomina clay was applied as a topical adjunct in 24 patients with Wagner grade 1 to 3 diabetic foot ulcers. Patients were stratified according to infection status. Outcomes included time to granulation tissue formation, epithelialization, complete ulcer closure, and safety. Multivariate analysis was performed to explore clinical and wound related factors associated with healing.

**Results:**

Fourteen patients presented with non infected ulcers and ten with clinically infected ulcers. Non infected ulcers demonstrated significantly faster healing trajectories and higher closure rates compared with infected ulcers. Median time to complete closure was shorter in the non infected group, and complete closure by week 20 was achieved in 92.9 percent of non infected ulcers compared with 40.0 percent of infected ulcers. Factors associated with delayed healing included age greater than 60 years, HbA1c greater than 7 percent, baseline wound size greater than 20 square centimeters, presence of infection, and higher Wagner grade. Adverse events were infrequent, mild, and transient, with no treatment discontinuations.

**Conclusion:**

Topical application of Dermomina clay was associated with favorable healing outcomes and a good safety profile, particularly in non infected diabetic foot ulcers. Although causal inference cannot be established, these findings support further evaluation of clay based therapies as low cost adjunctive interventions in diabetic foot care. Larger, controlled trials are warranted to confirm efficacy and define the role of Dermomina within evidence based management strategies for diabetic foot ulcers.

## Introduction

Dermomina clay is a naturally occurring mineral material composed predominantly of aluminosilicate phases, including illite, biotite, montmorillonite, kaolinite, muscovite, quartz, titania, calcite, microcline, and albite with calcium. Clays of this type arise from long term weathering and erosion of granitic and related rocks, resulting in fine grained materials with distinctive physicochemical properties that have supported medicinal and pharmaceutical applications for centuries (1).

The high specific surface area, ion exchange capacity, and sorptive properties of certain clay minerals make them attractive for use in pharmaceutical technology and dermopharmacy. These properties facilitate interaction with biological fluids, adsorption of exudates, and modulation of the local wound microenvironment (2). Experimental studies suggest that the antibacterial activity of some clays may be mediated by inorganic ions released from the mineral matrix in aqueous environments, generating conditions unfavorable for microbial survival. Proposed mechanisms include iron mediated redox reactions leading to hydroxyl radical formation, which can damage bacterial membranes and intracellular structures (3–6, 26).

Beyond intrinsic antibacterial activity, clay minerals have been investigated as platforms to enhance antimicrobial efficacy or as alternative strategies in the context of increasing antibiotic resistance (2, 7). *In vitro* and *in vivo* studies have demonstrated antibacterial effects of hydrated and reduced iron containing clays against a broad range of pathogens, with generally favorable safety profiles (1, 8).

A study conducted by Rosario Arias (9) concluded that alternative treatment with clay is feasible for achieving optimal healing in patients with ulcerative lesions. In addition, she observed that patients between the ages of 31 and 45 benefited the most and that clay therapy had no adverse effects.

Diabetic foot ulcers represent one of the most severe and costly complications of diabetes mellitus. Their pathogenesis is multifactorial, involving peripheral neuropathy, ischemia, structural foot deformities, and impaired immune responses, which together increase susceptibility to tissue breakdown and infection (10–12). Despite advances in multidisciplinary care, including debridement, antimicrobial therapy, and revascularization, outcomes remain suboptimal, particularly in infected ulcers (13, 14, 25).

Globally, diabetic foot ulcers are a leading cause of non traumatic lower limb amputations and are associated with substantial morbidity, mortality, and healthcare costs (15, 16). Epidemiological estimates indicate that fifteen to twenty five percent of individuals with diabetes will develop a foot ulcer during their lifetime, with markedly increased risk of amputation and reduced survival following ulcer related complications (13, 17). These burdens are particularly pronounced in low and middle income countries, where access to advanced wound care technologies and specialized services may be limited (18).

In this context, interest has grown in adjunctive or alternative therapies that may support wound healing, reduce infection related complications, and improve outcomes in diabetic foot care. Previous observational and experimental studies have evaluated the antibacterial and wound healing properties of clay based materials across different clinical scenarios, including inflammatory wounds, burns, venous ulcers, and chronic skin lesions. These studies have reported reductions in pain and inflammation, modulation of healing phases, and low rates of adverse effects following topical application (19–22, 27).

However, despite this body of literature, evidence specifically addressing the clinical use of medicinal clays in diabetic foot ulcers remains limited. In particular, there is a lack of studies characterizing healing dynamics in infected versus non infected ulcers, evaluating safety in real world clinical settings, and identifying patient and wound characteristics associated with favorable response. Recent integrative reviews have emphasized the need to explore alternative or adjunctive therapies in diabetic wound care, given persistently poor outcomes despite standard interventions (23).

Accordingly, the objective of the present study was to conduct a preliminary observational evaluation of Dermomina clay in patients with diabetic foot ulcers, with three specific aims. First, to describe healing trajectories and time dependent outcomes in infected and non infected ulcers. Second, to assess local safety and tolerability during topical application in routine clinical practice. Third, to explore clinical and wound related factors associated with ulcer closure using descriptive and multivariate analyses. By addressing these objectives, this study aims to generate foundational evidence to inform the design of adequately powered randomized controlled trials and to guide the rational development of clay based adjunctive therapies for diabetic foot ulcer management.

## Materials and methods

### Study design and setting

A pre-experimental, observational study was conducted to evaluate the clinical performance and safety of Dermomina^®^ clay as a topical adjunct in the management of infected and non-infected diabetic foot ulcers (DFUs). Patients were treated at the wound care units of Elio Fiallo Provincial Hospital (Pedernales, Dominican Republic) and the National Institute for Research on Infectious Diseases (INIEICONT) between March 2022 and October 2024.

### Study population

A total of 24 patients were recruited using convenience sampling. Eligible participants were adults with a diagnosis of diabetic foot ulcer classified as Wagner grade 1 to 3 who provided written informed consent. Exclusion criteria included incomplete treatment or follow-up, prior systemic antibiotic use before baseline evaluation, Wagner grade 4 or 5 ulcers, referral or admission to tertiary care hospitals, or death during the study period.

### Definition and classification of ulcer infection

Ulcer infection was defined as a pathological condition caused by the invasion and multiplication of microorganisms within host tissue, accompanied by tissue destruction and/or a host inflammatory response, in accordance with current international guidelines (14).

Clinical and microbiological criteria were jointly applied to classify ulcers as infected or non-infected using the International Working Group on the Diabetic Foot (IWGDF)/Infectious Diseases Society of America (IDSA) foot infection classification system. Baseline wound cultures were obtained in patients with clinically infected ulcers.

Patients classified as having infected ulcers received systemic antibiotic therapy according to institutional protocols, in combination with autolytic medical debridement, before and during topical Dermomina^®^ treatment.

### Assessment of vascular perfusion and neurological function

Lower-limb vascular perfusion was assessed using arterial and venous Doppler ultrasound to identify peripheral arterial disease and venous abnormalities. Neurological function was evaluated through standardized clinical examination performed by the neurology service, including assessment of protective sensation.

### Study material

Brown Dermomina^®^ clay was used throughout the study. Additional materials included sterile saline solution, potable water, a 1-L Pyrex glass container, a wooden spatula, sterile gauze, disposable gloves, elastic bandages, adhesive tape, disposable examination sheets, and a flexible measuring tape for wound assessment. Pain intensity was assessed using the Visual Analog Scale (VAS). The clay deposit from which Dermomina^®^ is sourced contains approximately two billion metric tons of material, with an estimated 23% clay content and a homogeneous mineral composition.

### Clay preparation and chemical characterization

Raw Dermomina^®^ clay underwent physical purification before clinical use. Coarse particulate matter and sand were removed by repeated decantation using natural spring water, allowing separation of the fine clay fraction based on sedimentation principles. The purified clay was stored at room temperature in plastic or glass containers, retaining its natural moisture content until application.

Chemical characterization of the purified clay was performed using X-ray fluorescence (XRF) analysis, as provided in the supplier’s Certificate of Analysis. XRF enables quantitative determination of major, minor, and trace elemental oxides in mineral materials.

As summarized in **Table 1**, Dermomina^®^ clay is predominantly composed of silica (SiO_2_) and alumina (Al_23_), consistent with an aluminosilicate mineral matrix. Minor oxides, including iron(III) oxide, sodium oxide, potassium oxide, magnesium oxide, and calcium oxide, were present at low concentrations, while trace elements were detected at minimal levels. Loss on ignition reflected structural water and volatile components, and total organic carbon and sulfur contents were negligible. This compositional profile confirms the mineral purity and chemical stability of the clay used in the study.

**Table 1:** Chemical composition of dermomina clay determined by x-ray fluorescence (XRF) dermomina laboratories (24).

Oxide / parameter	Chemical formula	Content (wt%)
Silicon dioxide	SiO_2_	50.60
Aluminum oxide	Al_23_	30.85
Iron(III) oxide	Fe_23_	3.02
Calcium oxide	CaO	0.28
Magnesium oxide	MgO	0.58
Sodium oxide	Na_2_O	1.18
Potassium oxide	K_2_O	1.94
Manganese oxide	MnO	0.10
Titanium dioxide	TiO_2_	0.30
Phosphorus pentoxide	P_25_	0.01
Chromium(III) oxide	Cr_23_	0.008
Barium (as Ba)	Ba	0.08
Loss on ignition (LOI)	—	11.54
**Total oxides**	—	100.5
Total organic carbon (TOC)	—	0.09
Total sulfur (TS)	—	<0.02

Acme Analytical Laboratories (Vancouver) Ltd. 1020 Cordova St. East, Vancouver, BC V6A 4A3, Canada Acme Analytical Laboratories (Vancouver) Ltd.

Bold values indicate statistically significant results (p < 0.05).

### Treatment protocol

Pain intensity was assessed using a 10-point Visual Analog Scale (0 = no pain; 10 = worst pain imaginable). Clinical data were collected using an 83-item validated questionnaire.

The Dermomina^®^ application protocol followed a standardized procedure:

- Dermomina^®^ clay was mixed with purified reverse-osmosis water in a glass container until a homogeneous paste was obtained.
- The ulcer was cleansed with sterile saline solution and chlorhexidine antiseptic soap.
- Sterile gauze was placed directly over the cleaned ulcer surface.
- A 2–3 mm layer of clay was applied over the gauze and secured with an elastic bandage.
- The poultice was removed daily; the wound was washed with warm or slightly alkaline water, and the application was repeated until predefined healing indicators were observed, including granulation tissue formation, reduction of wound margins, normalization of wound color, epithelialization, and complete closure.

### Outcome measures and wound assessment

Primary outcomes included time to granulation tissue formation, time to epithelialization, time to complete ulcer closure, weekly wound size reduction rate, and the proportion of ulcers achieving complete closure by week 20.

Ulcer size was assessed using a flexible measuring tape to measure the maximum diameter of the lesion at each evaluation. This method was applied consistently throughout follow-up to allow standardized longitudinal comparison of wound dimensions. Secondary outcomes included adverse events, microbiological findings in infected ulcers, and identification of independent predictors of wound closure. Healing progression was documented through clinical evaluation and serial photographic records.

### Safety assessment

Patients were actively monitored for adverse effects potentially associated with Dermomina^®^ application, including local irritation, inflammation, allergic reactions, or signs of systemic hypersensitivity. Adverse events were classified according to severity, duration, and requirement for treatment discontinuation.

### Statistical analysis

Data were entered into Microsoft Excel (version 8.0) and analyzed using Epi Info version 7 (Centers for Disease Control and Prevention, Atlanta, GA, USA). Data quality control included error correction and variable recoding before analysis. Continuous variables were summarized as mean ± standard deviation or median (range), as appropriate. Categorical variables were expressed as frequencies and percentages with 95% confidence intervals.

Normality of continuous variables was assessed using the Shapiro–Wilk test. Between-group comparisons were performed using the Mann–Whitney U test for continuous variables and chi-square or Fisher’s exact test for categorical variables. Multivariate logistic regression analysis was conducted to identify independent predictors of complete wound closure. Statistical significance was defined as p < 0.05.

### Ethical considerations

The study was conducted in accordance with the Declaration of Helsinki and was approved by the INIEICONT Ethics Committee. Written informed consent was obtained from all participants before study inclusion. Participants in the study gave their consent for the use of images and personal data as necessary.

## Results

### Material characterization

The chemical composition of Dermomina^®^ clay used in this study is presented in **Table 1**. X-ray fluorescence analysis demonstrated that the material was predominantly composed of silica (SiO_2_, 50.60 wt%) and alumina (Al_23_, 30.85 wt%), consistent with an aluminosilicate mineral matrix. Minor oxides, including iron(III) oxide, sodium oxide, potassium oxide, magnesium oxide, and calcium oxide, were present at low concentrations. Loss on ignition was 11.54 wt%, reflecting structural water and volatile components. Total organic carbon and sulfur content were negligible (<0.1 wt%), confirming the mineral purity and compositional stability of the clay applied during treatment.

### Statistical assumptions and analytical strategy

Normality of continuous variables was assessed using the Shapiro–Wilk test (**Table 2**). All variables demonstrated distributions consistent with normality (p > 0.05). Nevertheless, given the small sample size (n = 24) and the presence of unequal baseline characteristics between groups, non-parametric statistical methods (Mann–Whitney U test) were used for between-group comparisons as a conservative analytical approach.

**Table 2:** Shapiro-wilk normality test results.

Variable	Group	n	Mean ± SD	W	p-value	Interpretation
Time to complete closure (weeks)	Non-infected	14	12.1 ± 1.8	0.9441	0.4732	Normal
Time to complete closure (weeks)	Infected	10	17.4 ± 1.8	0.9496	0.6632	Normal
Weekly size reduction rate (%)	Non-infected	14	12.2 ± 0.8	0.9507	0.5722	Normal
Weekly size reduction rate (%)	Infected	10	6.5 ± 0.8	0.9931	0.9993	Normal

### Study population and baseline characteristics

A total of 24 patients with chronic diabetic foot ulcers were included in the analysis. At baseline, 14 patients (58.3%, 95% CI: 36.6%–77.9%) presented without clinical signs of infection, whereas 10 patients (41.7%, 95% CI: 22.1%–63.4%) had clinically infected ulcers (**Table 3**).

**Table 3:** Baseline clinical and demographic characteristics.

Characteristic	Non-infected (n=14)	Infected (n=10)	Difference [95% CI]	p-value
Age (years)	53.8 ± 11.9	62.4 ± 13.8	-8.6 [-18.2 to 1.1]	0.042*
Gender (male/female)	10/4	7/3	—	0.892†
BMI (kg/m²)	27.8 ± 5.2	29.1 ± 5.8	-1.3 [-5.9 to 3.3]	0.554‡
Diabetes duration (years)	11.2 ± 6.8	13.6 ± 7.2	-2.4 [-8.5 to 3.8]	0.385‡
HbA1c (%)	6.8 ± 1.9	7.6 ± 2.1	-0.8 [-2.2 to 0.6]	0.038*
Baseline wound size (cm²)	15.4 [5-42]	28.6 [8-156]	median difference: -13.2	0.003†
Peripheral neuropathy, n (%)	11 (78.6%, 95% CI: 49.2%-95.3%)	8 (80.0%, 95% CI: 44.4%-97.5%)	—	0.928†
Peripheral arterial disease, n (%)	8 (57.1%, 95% CI: 28.9%-82.3%)	7 (70.0%, 95% CI: 34.8%-93.3%)	—	0.510†

†Fisher's exact test; ‡Mann-Whitney U test; *Statistically significant at p < 0.05.

Baseline demographic and clinical characteristics are summarized in **Table 3**. Patients with infected ulcers were older than those without infection (mean age 62.4 vs. 53.8 years, p = 0.042) and had significantly larger baseline wound areas (median 28.6 cm² vs. 15.4 cm², p = 0.003). Glycemic control, body mass index, diabetes duration, sex distribution, peripheral neuropathy prevalence, and peripheral arterial disease prevalence did not differ significantly between groups.

### Baseline ulcer severity

Ulcer severity at baseline, assessed using the Wagner classification, differed significantly between groups (**Table 4**). Infected ulcers were more frequently classified as higher Wagner grades compared with non-infected ulcers (p = 0.015). Grade 3 ulcers accounted for 20.0% of infected wounds versus 7.1% of non-infected wounds, while Grade 1–2 ulcers predominated in both groups.

**Table 4:** Wagner grade distribution at baseline.

Wagner grade	Non-infected (n=14)	% [95% CI]	Infected (n=10)	% [95% CI]	p-value
Grade 1	6	42.9% [17.7%-71.4%]	3	30.0% [6.7%-65.3%]	0.015*
Grade 2	7	50.0% [23.0%-77.0%]	5	50.0% [18.7%-81.3%]	
Grade 3	1	7.1% [0.2%-33.9%]	2	20.0% [2.5%-55.6%]	

Statistical Test: Fisher's exact test.

### Time-to-event healing outcomes

Time-to-event healing outcomes are reported in **Table 5**. Non-infected ulcers demonstrated significantly shorter times to initial size reduction, granulation tissue formation, epithelialization, and complete closure compared with infected ulcers (all p < 0.001). By week 20, complete ulcer closure was achieved in 92.9% of non-infected ulcers compared with 40.0% of infected ulcers (p = 0.009).

**Table 5:** Time-to-event outcomes with 95% confidence intervals.

Parameter	Non-infected (n=14)	95% CI	Infected (n=10)	95% CI	p-value*
Time to first size reduction (weeks)	2.1	[1.8-2.6]	3.4	[2.8-4.2]	< 0.001
Time to complete closure (weeks)	12.1 ± 1.8	[11.1-13.2]	17.4 ± 1.8	[16.1-18.6]	< 0.001
Time to granulation tissue (weeks)	1.8	[1.2-2.4]	3.2	[2.6-4.1]	< 0.001
Time to epithelialization (weeks)	10.8	[8.2-12.6]	15.4	[13.2-17.8]	< 0.001
**Closure achieved by study end**	13/14 (92.9%)	[68.5%-98.7%]	4/10 (40.0%)	[16.8%-68.7%]	0.009†
**Surface area reduction at week 20 (%)**	94.8 ± 8.2	[90.1%-99.5%]	52.6 ± 24.8	[35.2%-70.0%]	< 0.001

*Mann-Whitney U test (two-tailed); †Fisher's exact test.

Bold values indicate statistically significant results (p < 0.05).

Surface area reduction at week 20 was significantly greater in the non-infected group (mean 94.8% vs. 52.6%, p < 0.001). Confidence intervals were narrower among non-infected ulcers, indicating less variability in healing trajectories.

### Wound healing dynamics

Detailed wound healing kinetics are presented in **Table 6**. The weekly wound size reduction rate was significantly higher in non-infected ulcers than in infected ulcers (12.2% vs. 6.5%, p < 0.001). Non-infected ulcers also exhibited significantly shorter times to granulation tissue formation, resolution of inflammation, and complete epithelialization (all p < 0.001).

**Table 6:** Wound healing dynamics with 95% confidence intervals.

Parameter	Non-infected (n=14)	95% CI	Infected (n=10)	95% CI	p-value*
**Weekly size reduction rate (%)**	12.2 ± 0.8	[11.7%-12.7%]	6.5 ± 0.8	[5.9%-7.1%]	< 0.001
Time to granulation tissue (weeks)	1.8 ± 0.6	[1.5-2.2]	3.2 ± 0.7	[2.7-3.7]	< 0.001
Resolution of inflammation (weeks)	2.2 ± 0.8	[1.8-2.8]	4.6 ± 0.9	[4.0-5.2]	< 0.001
Complete epithelialization (weeks)	10.8 ± 1.2	[10.1-11.5]	15.4 ± 1.5	[14.5-16.3]	< 0.001

*Mann-Whitney U test (two-tailed).

Bold values indicate statistically significant results (p < 0.05).

### Temporal evolution of wound healing

The temporal evolution of wound healing outcomes is shown in **Table 7**. At week 4, partial or complete healing was observed in 85.7% of non-infected ulcers compared with 50.0% of infected ulcers. By week 8, complete closure occurred in 57.1% of non-infected ulcers and 20.0% of infected ulcers (p = 0.015). At week 20, complete closure was achieved in 92.9% of non-infected ulcers versus 40.0% of infected ulcers (p < 0.001).

**Table 7:** Temporal evolution of wound healing.

Time point	Non-infected (n=14)	% [95% CI]	Infected (n=10)	% [95% CI]	p-value*
Week 4	4 complete	28.6% [8.4%-58.1%]	1 complete	10.0% [0.3%-44.5%]	0.236
	8 partial	57.1% [28.3%-82.3%]	4 partial	40.0% [12.2%-73.8%]	
	2 no change	14.3% [1.8%-42.8%]	5 no change	50.0% [18.7%-81.3%]	
Week 8	8 complete	57.1% [28.3%-82.3%]	2 complete	20.0% [2.5%-55.6%]	0.015*
	5 partial	35.7% [12.8%-64.9%]	4 partial	40.0% [12.2%-73.8%]	
	1 no change	7.1% [0.2%-33.9%]	4 no change	40.0% [12.2%-73.8%]	
Week 20	13 complete	92.9% [68.5%-98.7%]	4 complete	40.0% [16.8%-68.7%]	< 0.001
	1 partial	7.1% [0.2%-33.9%]	4 partial	40.0% [12.2%-73.8%]	
	0 no change	—	2 no change	20.0% [2.5%-55.6%]	

*Fisher's exact test.

### Multivariate predictors of complete ulcer closure

Results of the multivariate logistic regression analysis are summarized in **Table 8**. Independent predictors of reduced odds of complete ulcer closure included higher Wagner grade, larger initial wound size (>20 cm²), presence of infection, HbA1c > 7%, age > 60 years, and peripheral arterial disease (all p < 0.05). Wagner Grade 3 demonstrated the strongest negative association with closure (adjusted OR = 0.15, 95% CI: 0.06–0.38).

**Table 8:** Multivariate logistic regression: predictors of complete wound closure.

Factor	Adjusted OR	95% CI	p-value
Age > 60 years	0.58	[0.35-0.96]	0.042*
HbA1c > 7%	0.52	[0.31-0.87]	0.038*
Initial wound size > 20 cm²	0.38	[0.20-0.72]	0.003*
Presence of infection (yes vs. no)	0.41	[0.22-0.76]	0.008*
Wagner Grade 2 (vs. Grade 1)	0.48	[0.28-0.82]	0.012*
Wagner Grade 3 (vs. Grade 1)	0.15	[0.06-0.38]	< 0.001*
Peripheral arterial disease (yes vs. no)	0.62	[0.38-0.94]	0.036*

*p < 0.05 (statistically significant) OR = Odds Ratio; CI = Confidence Interval.

### Safety outcomes

Safety outcomes are presented in **Table 9**. Two mild adverse events were reported, yielding an overall incidence of 8.3% (95% CI: 1.0%–27.0%). These events consisted of transient local discomfort or mild skin irritation and resolved within approximately two days. No serious adverse events, treatment discontinuations, amputations, or deaths were observed during follow-up.

**Table 9:** Adverse events with incidence rates and 95% CI.

Adverse Event Type	Non-infected (n=14)	Rate [95% CI]	Infected (n=10)	Rate [95% CI]	Total (n=24)	Rate [95% CI]
Total AE events	1	7.1% [0.2%-33.9%]	1	10.0% [0.3%-44.5%]	2	8.3% [1.0%-27.0%]
Mild discomfort	1	7.1% [0.2%-33.9%]	0	—	1	4.2% [0.1%-21.4%]
Temporary skin irritation	0	—	1	10.0% [0.3%-44.5%]	1	4.2% [0.1%-21.4%]
Treatment discontinuation	0	0% [0%-23.2%]	0	0% [0%-30.9%]	0	0% [0%-14.2%]
Duration of events (days)	2.1 ± 0.8	[1.3-2.9]	2.4 ± 0.6	[1.9-2.9]	2.2 ± 0.7	[1.6-2.8]

### Microbiological findings

Microbiological findings from first wound cultures are summarized in **Table 10**. Pseudomonas aeruginosa was the most frequently isolated pathogen (25.6%), followed by Staphylococcus aureus and Escherichia coli. Other Gram-negative organisms, including extended-spectrum β-lactamase–producing E. coli and Stenotrophomonas maltophilia, were identified at lower frequencies.

**Table 10:** Microorganisms isolated in the first culture of ulcer patients treated at Elio Fiallo provincial hospital and the national institute of research on infectious diseases, 2022–2024.

Isolated microorganism	Frequency (n)	Percentage (%)
*Escherichia coli*	2	5.1
*Escherichia coli* (ESBL-producing)	1	2.6
*Klebsiella ozaenae*	1	2.6
*Pseudomonas aeruginosa*	10	25.6
Methicillin-resistant *Staphylococcus* spp. (MR)	2	5.1
*Staphylococcus aureus*	3	7.7
*Stenotrophomonas maltophilia*	1	2.6

Source. Primary data obtained from microbiological cultures performed at Elio Fiallo Provincial Hospital and the National Institute of Research on Infectious Diseases (2022–2024).

## Discussion

This pre-experimental observational study provides clinically relevant evidence on the use of Dermomina^®^ clay as a topical adjunct in the management of chronic diabetic foot ulcers (DFUs). By integrating quantitative outcomes, multivariate modeling, microbiological findings, and longitudinal visual documentation, the results collectively describe treatment response patterns, determinants of healing, and safety within a real-world endocrine complication setting.

### Differential Healing According to Infection Status

**Figure 1:**
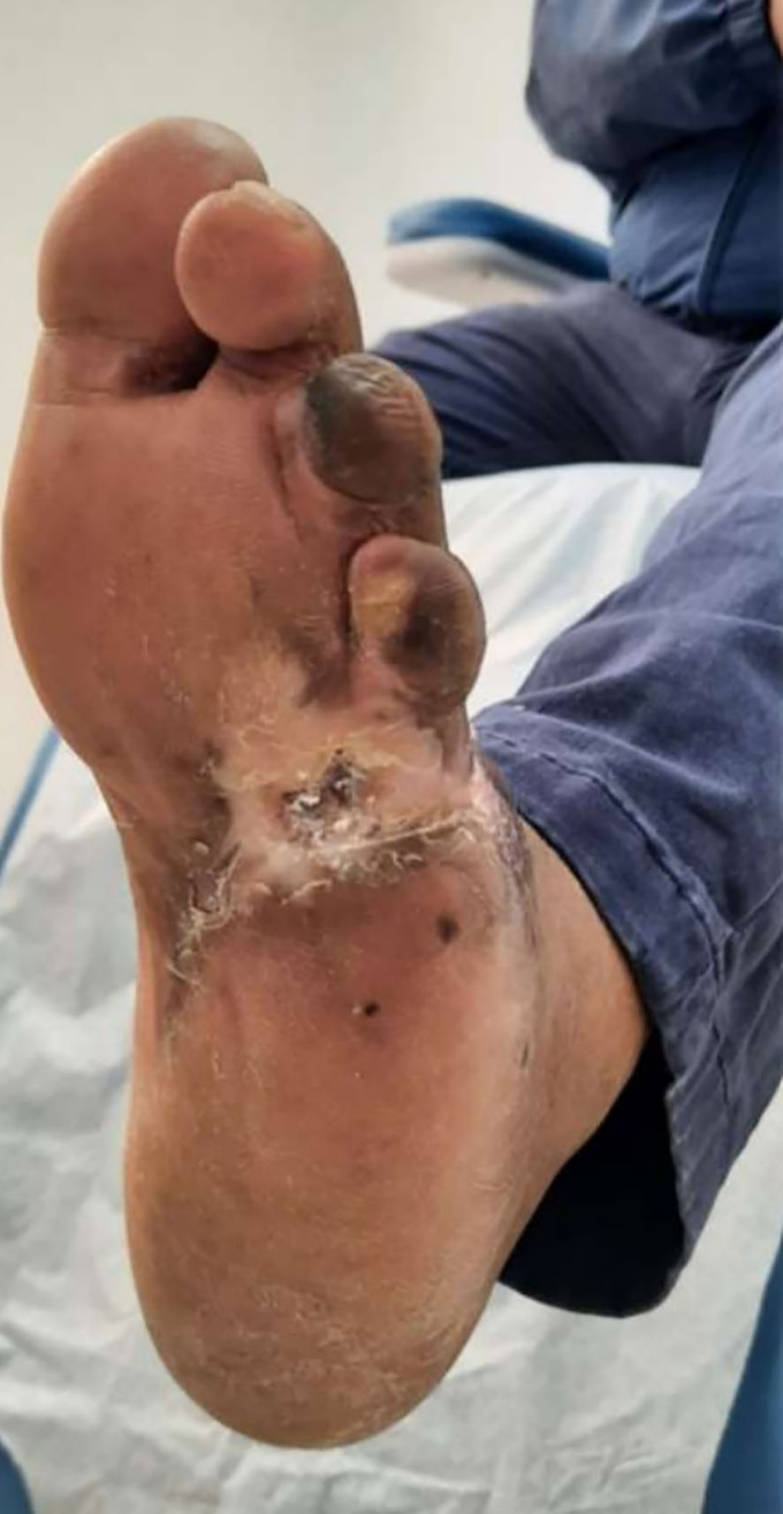
Time to wound healing in patients treated with Dermomina® (n=24). Comparison between non-infected and infected groups.

**Figure 2:**
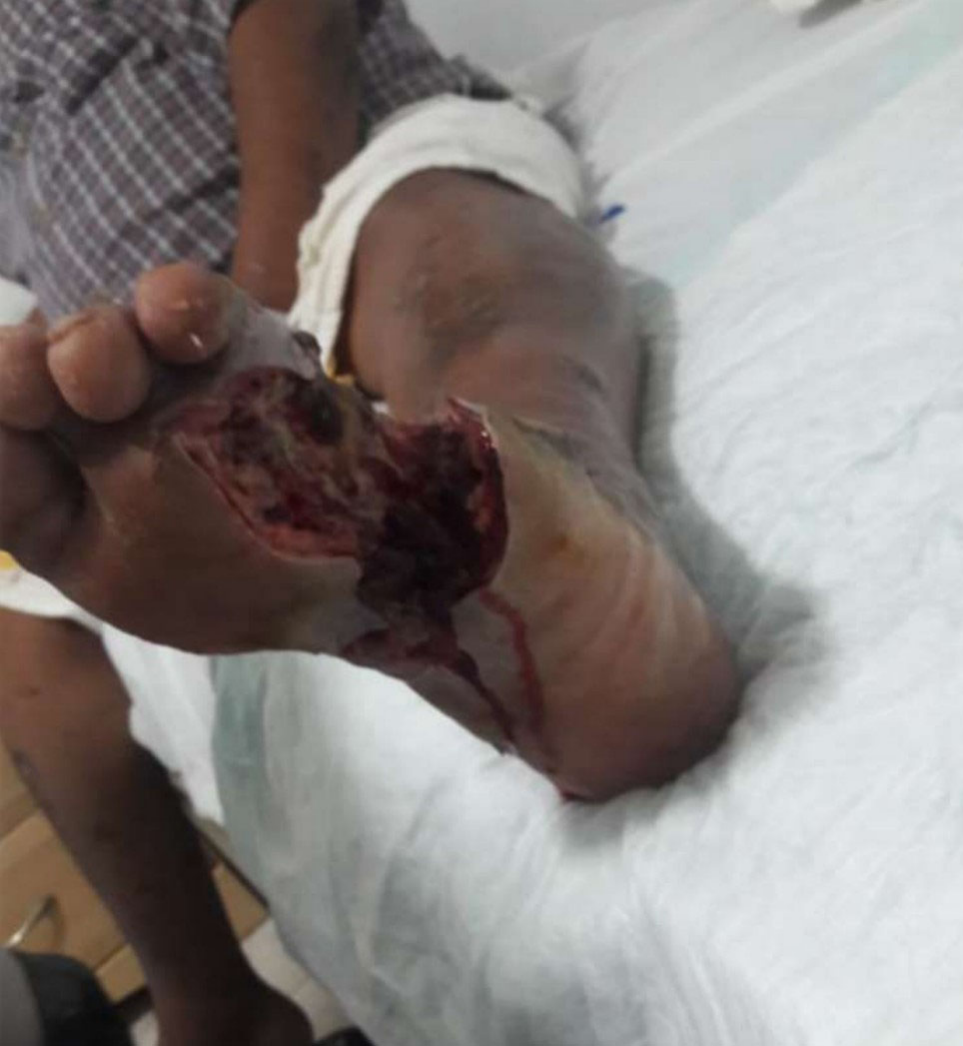
Wound healing dynamics in patients treated with Dermomina® (n=24). Comparison of granulation, size reduction, inflammation resolution, and epithelialization.

A consistent and robust finding across analyses was the markedly superior healing trajectory observed in non-infected ulcers compared with infected ulcers. Non-infected wounds demonstrated significantly shorter times to initial size reduction, granulation tissue formation, epithelialization, and complete closure (**Tables 5**, **6**), with these differences remaining evident throughout follow-up (**Table 7**). These findings are visually reinforced by the divergence in healing curves shown in **Figure 1**, where non-infected ulcers exhibited faster progression to closure, and by **Figure 2**, which illustrates parallel acceleration across multiple wound healing domains.

This pattern aligns with established pathophysiological understanding that infection and bacterial burden disrupt wound healing through sustained inflammation, protease activity, and impaired angiogenesis. Importantly, while the present study does not permit causal inference, the association between non-infected status and improved outcomes remained statistically significant after adjustment for relevant clinical covariates in multivariate analysis (**Table 8**).

### Wound healing dynamics and mechanistic considerations

The observed differences in weekly wound size reduction rate—nearly double in non-infected ulcers compared with infected ulcers (**Table 6**)—suggest that Dermomina^®^ application was associated with more favorable local wound dynamics in the absence of active infection. Earlier granulation and faster resolution of inflammation further support a potential role of the clay environment in promoting conditions conducive to tissue repair.

The chemical characterization of Dermomina^®^ (**Table 1**) confirms a predominantly aluminosilicate composition with minimal organic content, supporting hypotheses related to moisture regulation, adsorption of exudate, and modulation of the wound microenvironment. While mechanistic pathways were not directly assessed in this study, the convergence of faster granulation and epithelialization observed in **Figures 1** and **2** suggests a biologically plausible interaction between wound status and topical clay application.

### Influence of baseline clinical and wound characteristics

Baseline characteristics significantly influenced healing outcomes. Larger initial wound size (>20 cm²), higher Wagner grade, presence of infection, suboptimal glycemic control (HbA1c >7%), advanced age, and peripheral arterial disease were all independently associated with reduced odds of complete closure (**Table 8**). Among these, wound severity emerged as the strongest predictor, with Wagner Grade 3 ulcers exhibiting substantially lower closure probability.

These findings underscore the importance of early intervention. Smaller, less severe ulcers demonstrated more consistent and rapid healing trajectories, suggesting that Dermomina^®^ may be most effective when applied before advanced tissue damage and infection are established. This has direct clinical implications for endocrinology practice, where early identification and intervention in DFUs are critical to preventing progression, hospitalization, and amputation.

### Visual documentation and clinical heterogeneity

**Figure 3:**
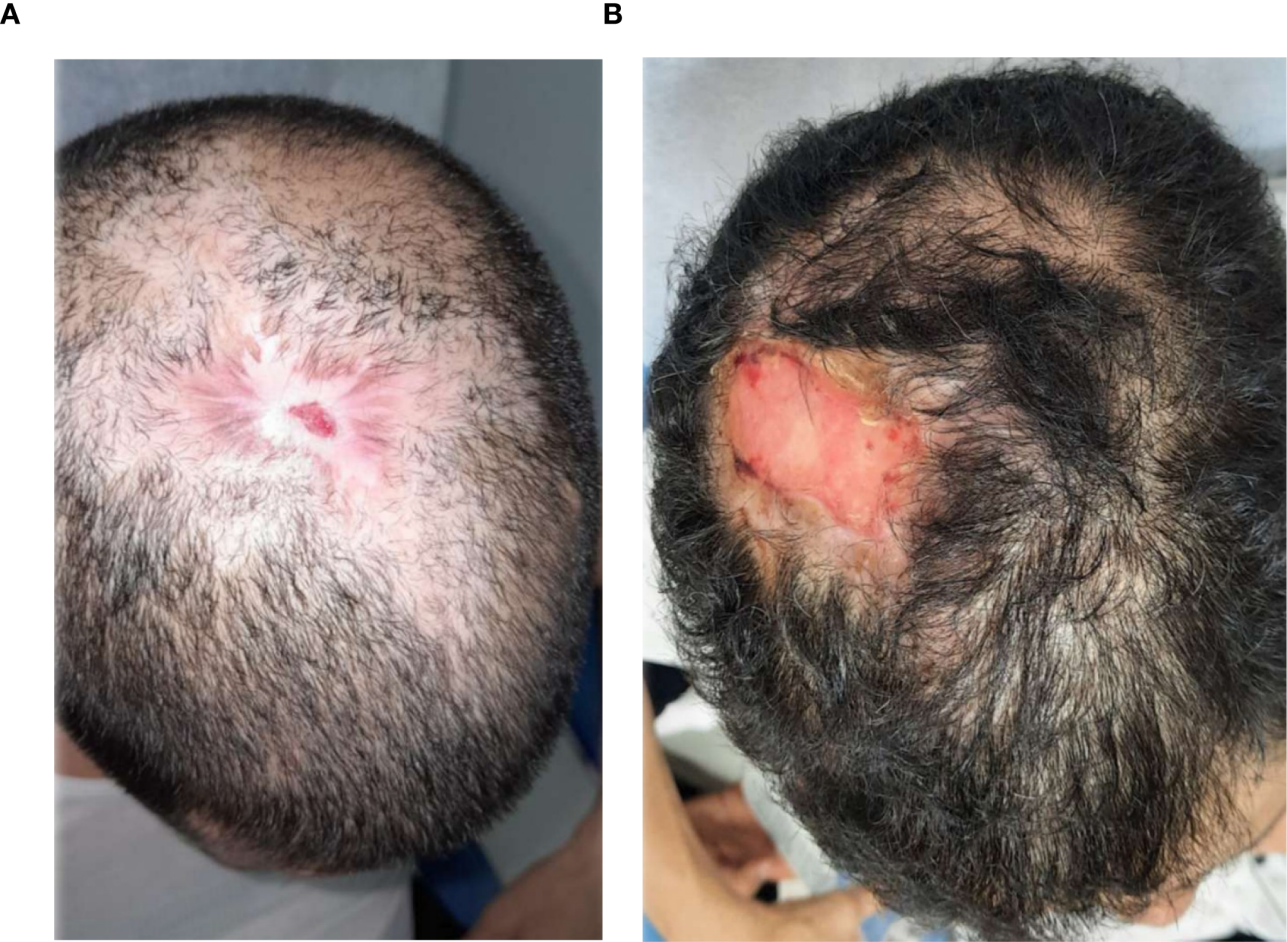
**(A)** Patient 1, male, 43 years, Wagner grade 4, dorsoplantar ulcer. **(B)** Complete epithelialization after 38 weeks.

**Figure 4:**
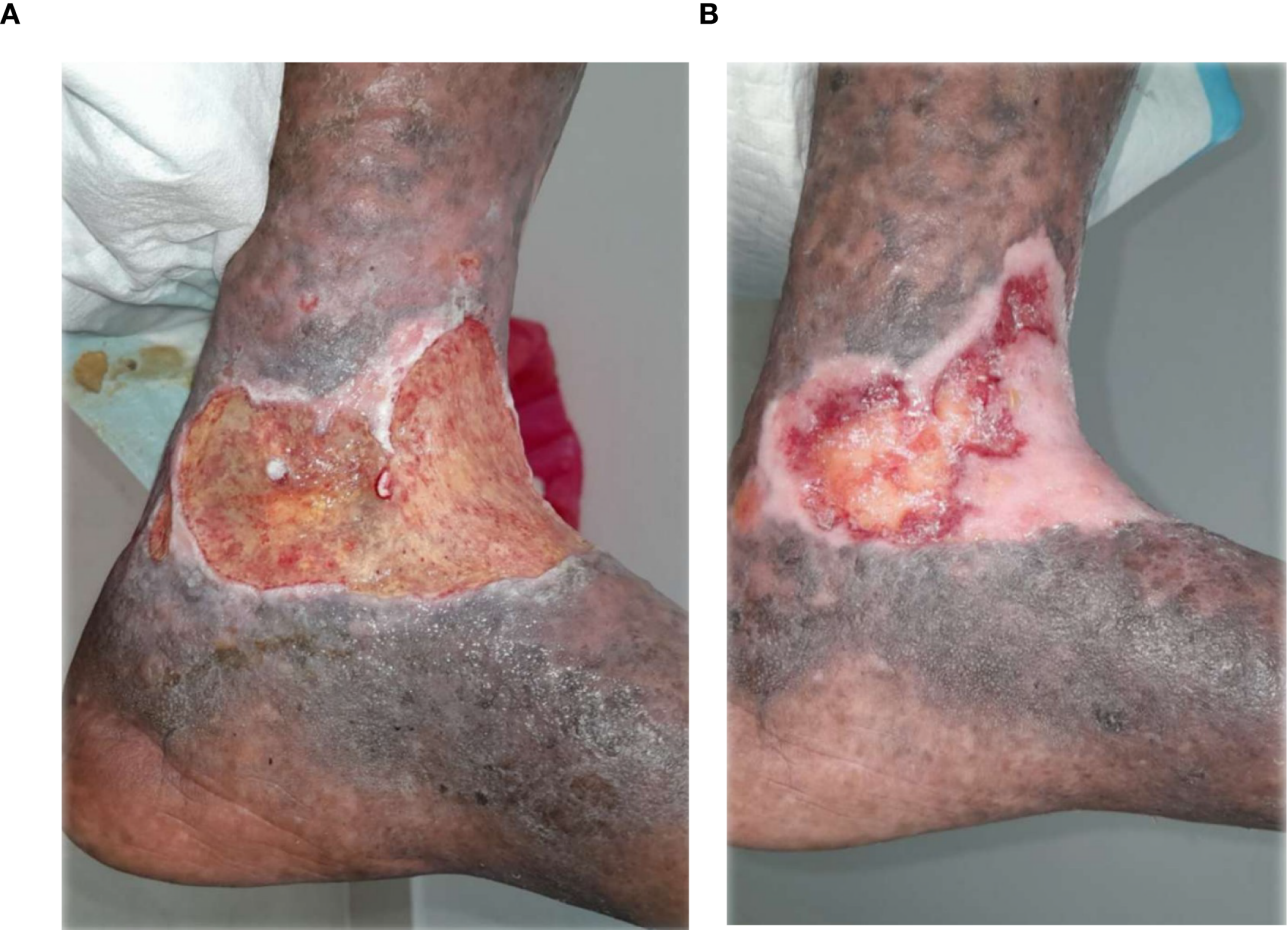
**(A)** Patient 2, male, 45 years, scalp abscess. **(B)** >95% epithelialization after 27.4 weeks.

**Figure 5:**
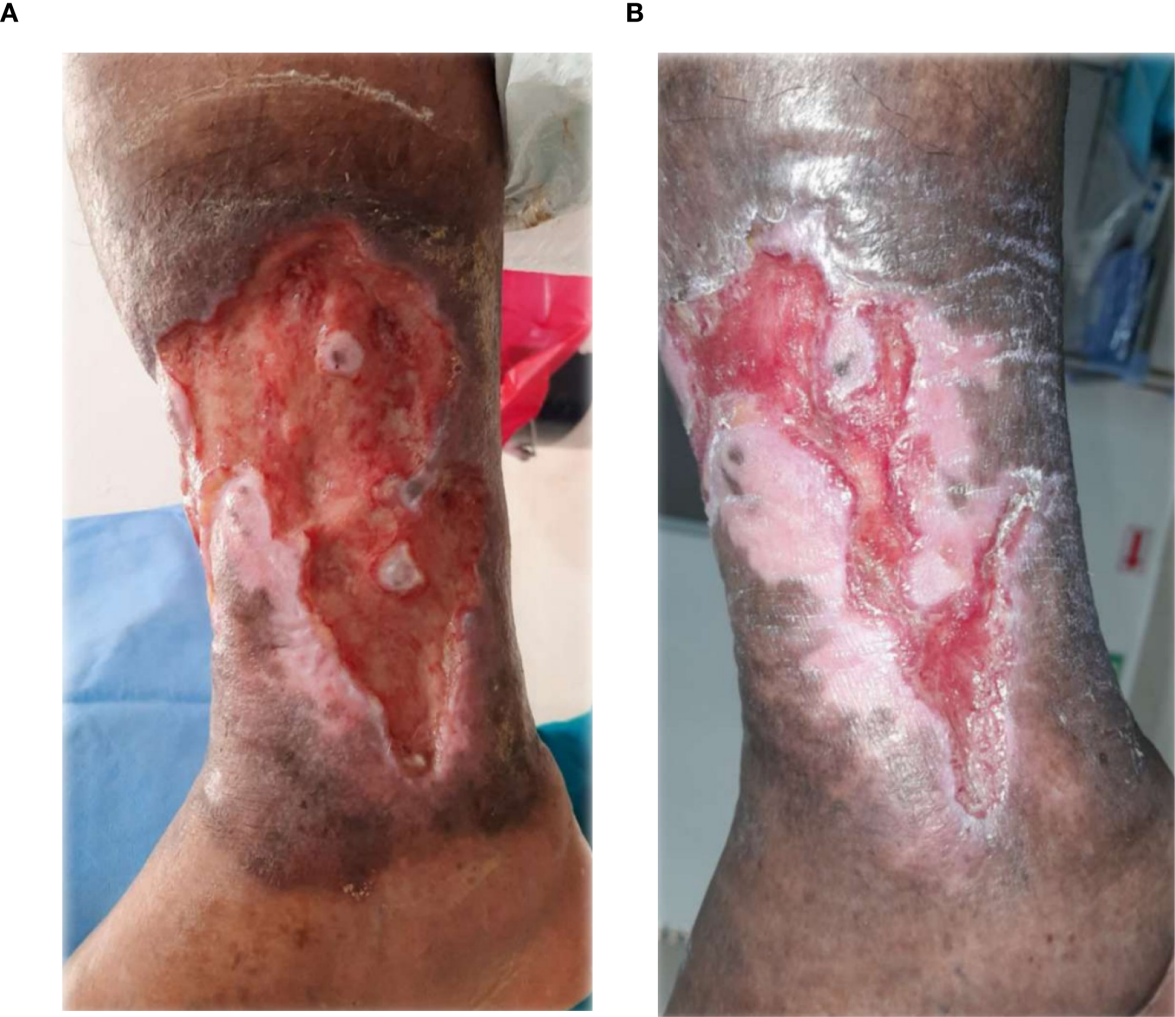
**(A)** Patient 3, male, 70 years, vascular ulcer. **(B)** ~60% epithelialization after 35.2 weeks.

**Figure 6:**
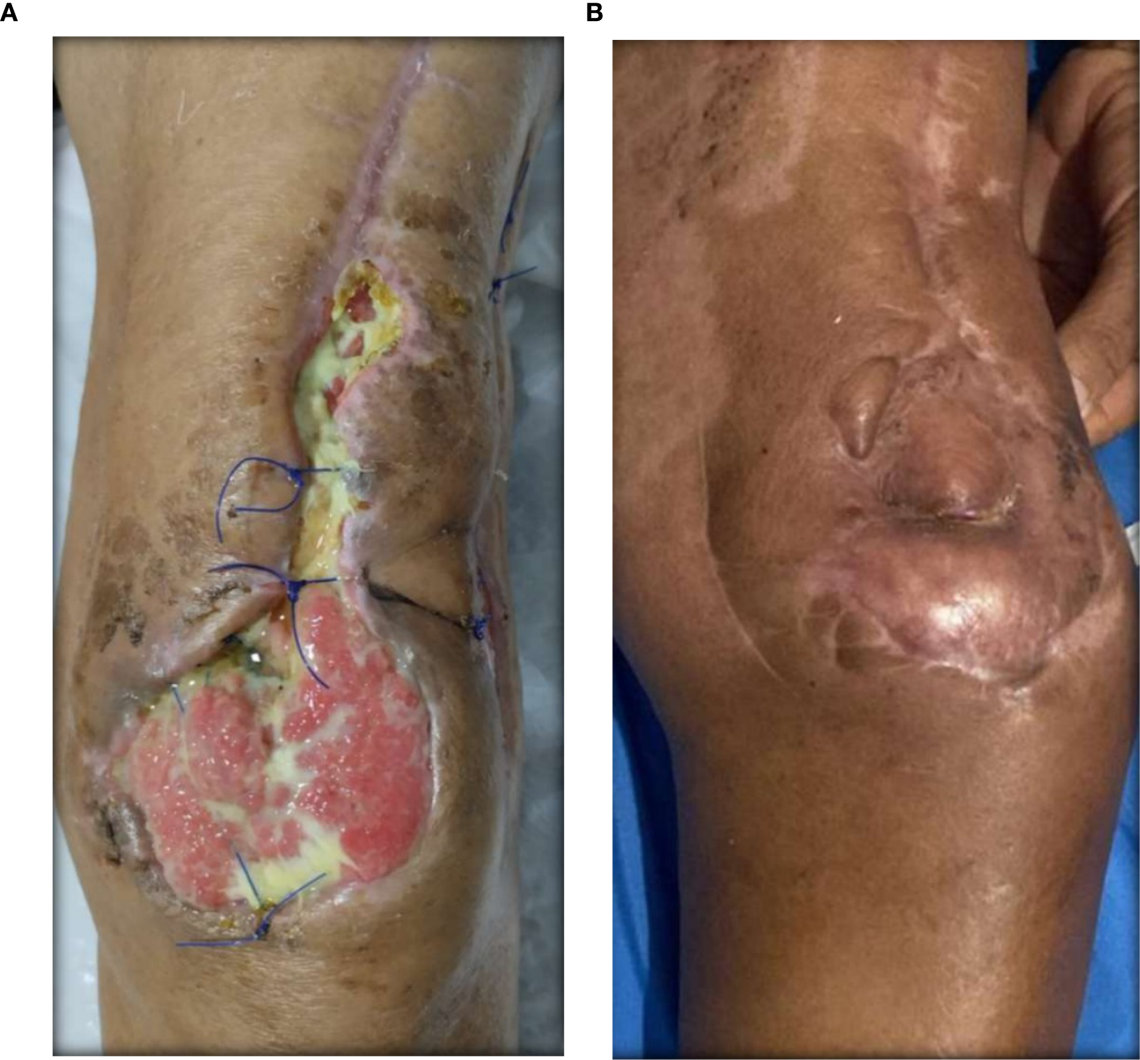
**(A)** Patient 4, male, 50 years, vascular ulcers. **(B)** ~75% epithelialization after 34.6 weeks.

**Figure 7:**
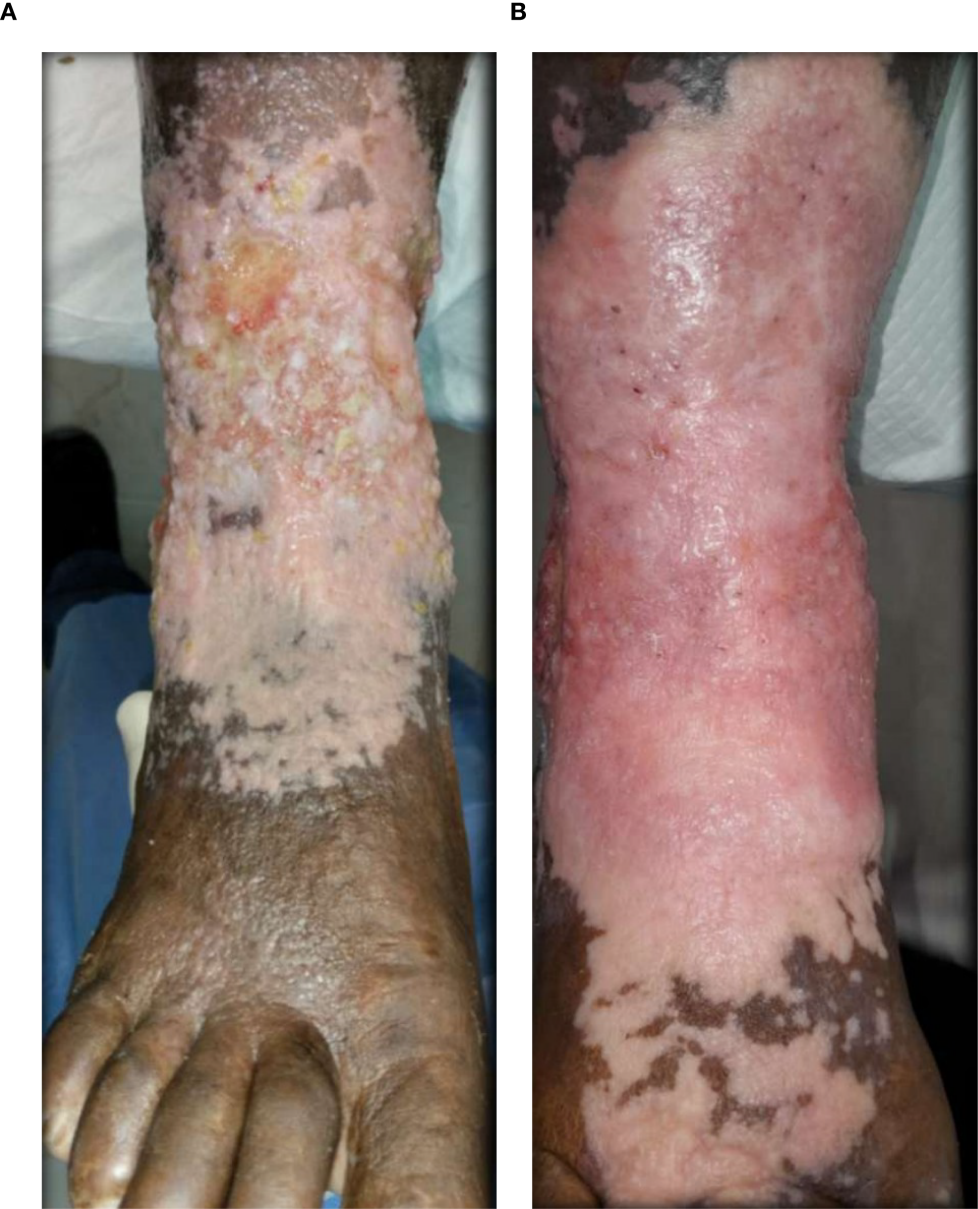
**(A)** Patient 5, female, 72 years, infected ulcer. **(B)** Active granulation without infection after 25 weeks.

**Figure 8:**
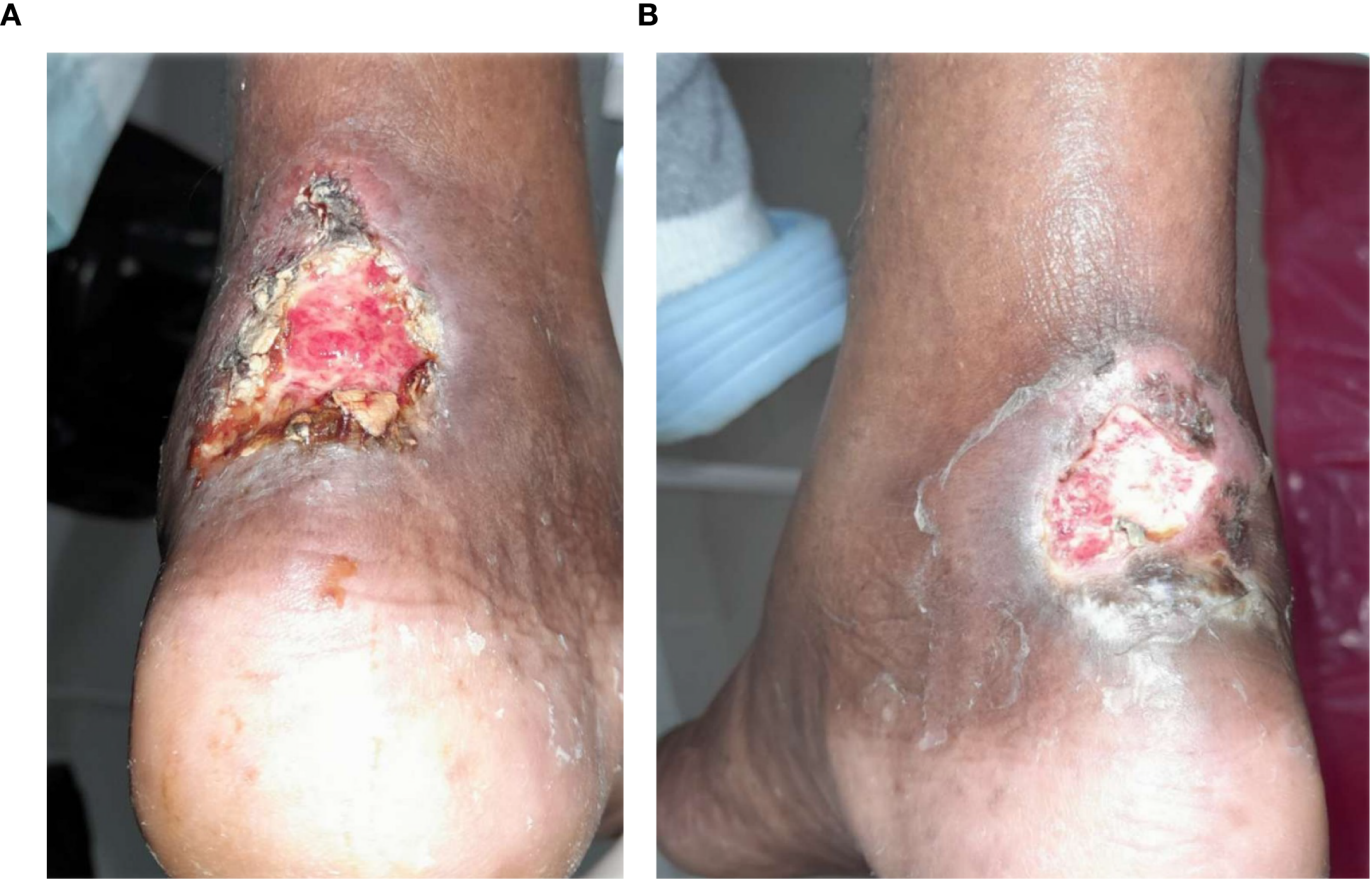
**(A)** Patient 8, female, 60 years, vascular ulcer. **(B)** ~80% epithelialization after 26 weeks.

**Figure 9:**
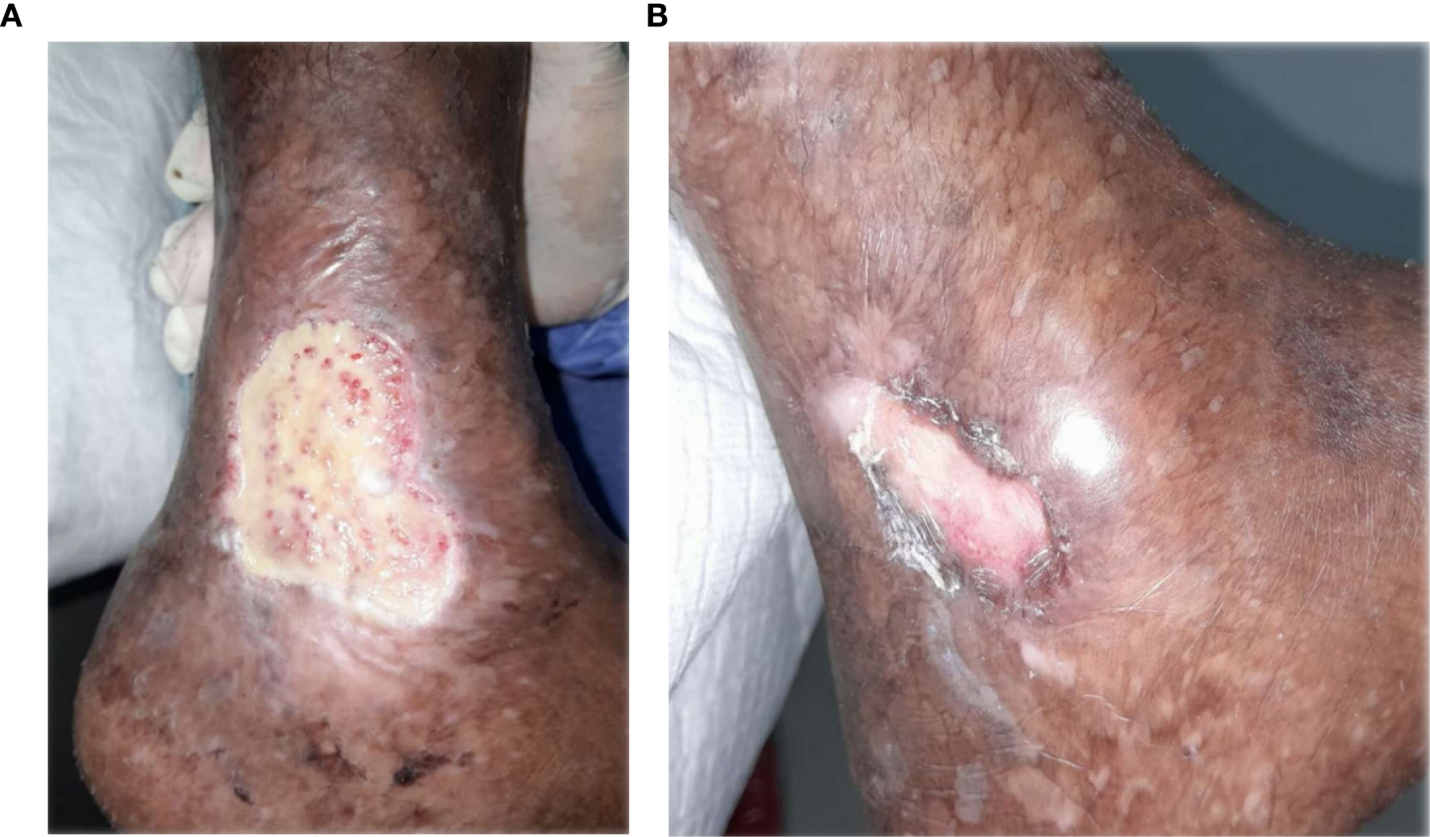
**(A)** Patient 14, female, 67 years, Achilles-tendon abscess. **(B)** Remission after 0.5 weeks.

**Figure 10:**
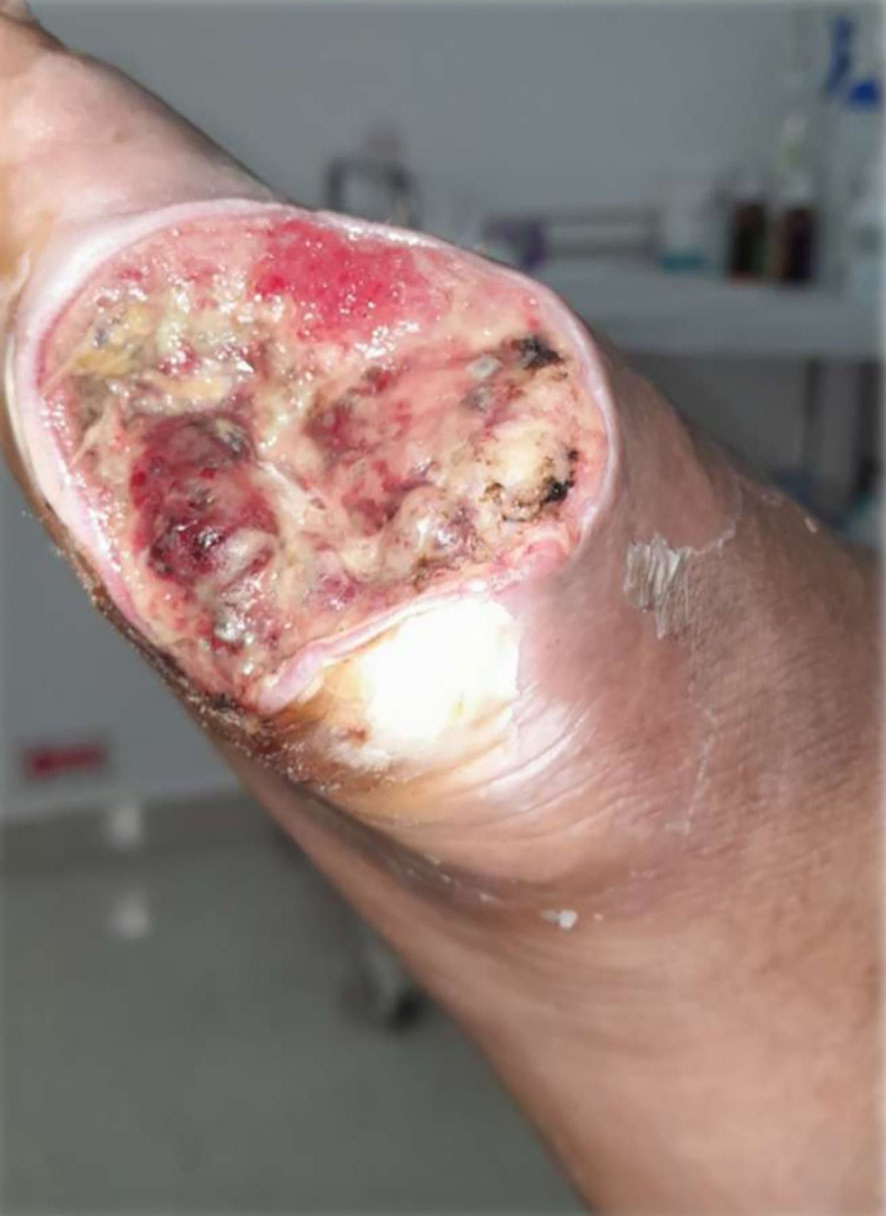
**(A)** Patient 16, male, 42 years, malleolar ulcer. **(B)** Active granulation after 23.1 weeks.

**Figure 11:**
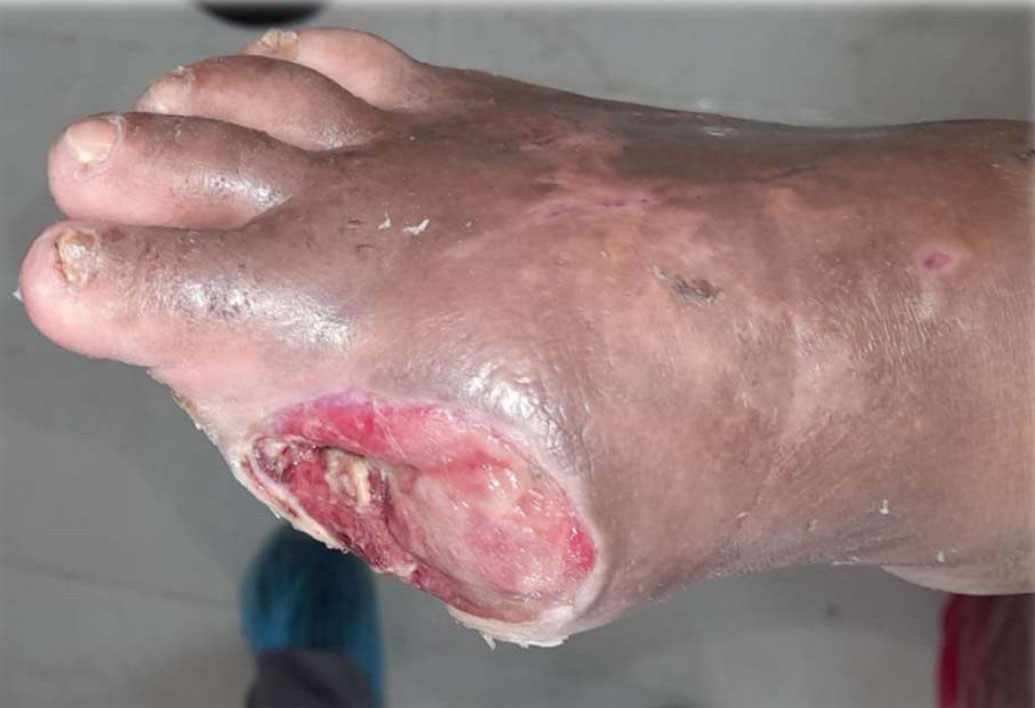
**(A)** Patient 18, female, 32 years, abscess at surgical bed. **(B)** Granulation phase after 1.5 weeks.

The serial clinical images (**Figures 3**–**11**) provide qualitative corroboration of the quantitative findings and highlight the heterogeneity of wound presentations and responses. Notably, meaningful healing responses were observed across a range of ulcer types, including infected ulcers, vascular ulcers, surgical bed abscesses, and complex anatomical locations.

Several cases illustrate substantial epithelialization or granulation despite advanced age, vascular disease, or infection (e.g., **Figures 7**-**10**), reinforcing that while baseline factors modulate the probability of closure, clinically relevant improvement may still occur. These images also reflect the real-world variability inherent to DFU management and support the external validity of the findings.

### Microbiological context

Microbiological analysis identified Pseudomonas aeruginosa as the most frequently isolated pathogen, followed by Staphylococcus aureus and Escherichia coli (**Table 10**). The predominance of Gram-negative organisms, including non-fermenters, is consistent with chronic DFU microbiology and may partially explain the attenuated healing observed in infected ulcers. Importantly, no microbiological worsening or superinfection attributable to Dermomina^®^ application was observed.

### Safety and tolerability

Dermomina^®^ demonstrated a favorable safety profile. Only two mild, transient adverse events were reported, both resolving without treatment discontinuation (**Table 9**). Similar to the study by Rosario Arias (9) no serious adverse events, amputations, or deaths occurred. These findings support the local tolerability of the intervention and are particularly relevant given the vulnerability of patients with diabetes-related wounds.

### Strengths and limitations

The strengths of this study include comprehensive outcome assessment, multivariate adjustment for key confounders, microbiological characterization, and longitudinal visual documentation. However, several limitations must be acknowledged. The study’s observational, pre-experimental design and small sample size limit causal inference and generalizability. The absence of a randomized control group precludes definitive attribution of outcomes to Dermomina^®^ alone, and unmeasured confounders cannot be excluded. Nevertheless, within these constraints, the consistency of findings across tables, figures, and clinical images strengthens confidence in the observed associations and supports further investigation.

### Clinical and research implications

Taken together, the findings suggest that Dermomina^®^ may represent a safe and potentially useful adjunct in the management of chronic DFUs, particularly in non-infected, early-to-moderate wounds with favorable baseline characteristics. The results also highlight the critical role of infection control and metabolic optimization in maximizing therapeutic response.

Future randomized controlled trials with larger sample sizes, standardized comparator treatments, and mechanistic endpoints are warranted to confirm efficacy, define optimal patient selection, and clarify biological mechanisms.

## Limitations

This preliminary observational study was designed to characterize healing patterns and identify potential safety signals associated with Dermomina clay therapy in diabetic foot ulcers. As such, several limitations inherent to its exploratory design must be acknowledged.

### Study design and scope

As a pre experimental observational cohort without randomization or a control group, this study cannot establish causality or directly compare the effectiveness of Dermomina with standard wound care. The concurrent use of nutritional supplementation, including ABINTRA, limits the ability to attribute observed healing exclusively to clay therapy. In addition, the single center design and convenience sampling from wound care clinics in the Dominican Republic restrict the generalizability of the findings to similar clinical and resource settings.

Baseline imbalances between infected and non infected groups were observed, including differences in age, glycemic control, and baseline wound size. These imbalances were addressed through multivariate adjustment; however, residual confounding cannot be excluded.

### Sample size and statistical considerations

The modest sample size of twenty four participants limits statistical power and increases uncertainty around effect estimates. More importantly, the multivariate regression model included a limited number of outcome events per variable, increasing the risk of model overfitting. Consequently, adjusted associations should be interpreted as descriptive within this cohort rather than as precise population estimates or predictive tools. The multivariate analysis was intended to identify candidate variables for future hypothesis testing rather than to confirm independent causal effects.

By contrast, univariate findings describing healing kinetics and closure proportions are less vulnerable to overfitting and provide more robust descriptive information regarding treatment response patterns in this population.

### Outcome assessment and missing comparisons

Clinical outcome assessment was not blinded and relied on pragmatic bedside measurements rather than objective planimetric or digital imaging techniques, introducing potential measurement bias. Furthermore, the absence of a parallel control or standard of care comparison group limits the ability to contextualize observed healing rates relative to expected outcomes with conventional topical therapies.

### Strengths and intended contribution

Despite these limitations, the study achieves its primary objective of establishing an initial safety and feasibility profile for Dermomina in diabetic foot ulcers. The low incidence of adverse events, all of which were mild and transient, supports local tolerability. Additionally, the detailed characterization of healing trajectories across infection status and ulcer severity provides essential descriptive data to inform the design of adequately powered randomized studies. Identification of infection status and Wagner grade as key determinants of outcome offers clinically relevant stratification variables for future trials.

## Conclusion

In this observational cohort, treatment with Dermomina clay was associated with favorable healing outcomes, particularly in non infected diabetic foot ulcers. Higher closure rates and faster healing dynamics were observed among patients who were younger, had better glycemic control, smaller baseline wound size, lower Wagner grade ulcers, and the absence of infection.

Although causal inference is not possible, the consistency of healing patterns across multiple clinical endpoints and the favorable safety profile suggest that Dermomina may represent a promising low cost adjunct in the management of diabetic foot ulcers. These findings are particularly relevant in resource-constrained settings where access to advanced wound care technologies is limited.

Importantly, this study establishes a foundation for further investigation rather than definitive evidence of efficacy. The data generated provide critical information to support the design of rigorous randomized controlled trials aimed at evaluating the clinical effectiveness of Dermomina under controlled conditions.

Future research should prioritize randomized allocation, blinded outcome assessment, comparison with standard wound care therapies, stratification by ulcer severity and infection status, and standardized nutritional protocols to isolate treatment effects. Adequately powered studies will be essential to confirm these preliminary observations and to define the role of Dermomina within evidence based diabetic foot ulcer management strategies.

## Data Availability

The raw data supporting the conclusions of this article will be made available by the authors, without undue reservation.
